# LY294002 and sorafenib as inhibitors of intracellular survival pathways in the elimination of human glioma cells by programmed cell death

**DOI:** 10.1007/s00441-021-03481-0

**Published:** 2021-07-08

**Authors:** Zając A, Sumorek-Wiadro J, Maciejczyk A, Langner E, Wertel I, Rzeski W, Jakubowicz-Gil J

**Affiliations:** 1 Department of Functional Anatomy and Cytobiology, Institute of Biological Sciences, Maria Curie-Sklodowska University, Lublin, Poland; 2 Department of Medical Biology, Institute of Agricultural Medicine, Lublin, Poland; 3 1st Department of Gynecology, University School of Medicine, Lublin, Poland

**Keywords:** Gliomas, LY294002, Sorafenib, Apoptosis, Autophagy

## Abstract

Gliomas are aggressive brain tumors with very high resistance to chemotherapy throughout the overexpression of multiple intracellular survival pathways. Therefore, the aim of the present study was to investigate for the first time the anticancer activity of LY294002, phosphatidylinositol 3-kinase (PI3K) inhibitor and sorafenib, and rapidly accelerated fibrosarcoma kinase (Raf) inhibitor in the elimination of human glioma cells by programmed cell death. MOGGCCM (anaplastic astrocytoma, III) and T98G (glioblastoma multiforme, IV) cell lines incubated with LY294002 and/or sorafenib were used in the experiments. Simultaneous treatment with both drugs was more effective in the elimination of cancer cells on the way of apoptosis with no significant necrotic effect than single application. It was correlated with decreasing the mitochondrial membrane potential and activation of caspase 3 and 9. The expression of Raf and PI3K was also inhibited. Blocking of those kinases expression by specific siRNA revealed significant apoptosis induction, exceeding the level observed after LY294002 and sorafenib treatment in non-transfected lines but only in MOGGCCM cells. Our results indicated that combination of LY294002 and sorafenib was very efficient in apoptosis induction in glioma cells. Anaplastic astrocytoma cells turned out to be more sensitive for apoptosis induction than glioblastoma multiforme after blocking PI3K and Raf expression with siRNA.

**Supplementary Information:**

The online version contains supplementary material available at 10.1007/s00441-021-03481-0.

## Introduction

Anaplastic astrocytoma (AA, WHO grade III) and glioblastoma multiforme (GBM, WHO grade IV) are the most malignant, primary brain cancers. They are characterized by aggressive growth, invasive infiltration into healthy brain tissue, and poor prognosis. In the case of AA, it means years while in GBM even couple of months. Standard treatment based on surgical resection followed by radio- and chemotherapy does not lead to cure and only prolongs the patient’s life. Removing the tumor during the surgery procedure is often complicated by its localization and necessary of some margin of healthy tissue disposal, which could be harmful in case of our brain tissue. In the last decades, there has been standard chemotherapy of malignant gliomas based on cytostatics with methylating properties, such as temozolomide, procarbazine, dacarbazine, or streptozotocine. Their alkylating activity should lead to elimination of the glioma cells upon the cell cycle arrest and apoptotic or autophagic cell death. Apoptosis is a defense mechanism that leads to elimination of transformed, infected, or damaged cells, and its induction causes to the activation of caspases (proteases—exist in cells by normal conditions as inactive pro-forms) especially 3, 8, and 9 which controls the proper running of this process. Autophagy, in turn, is a physiological process of damaged cell compartments recycling, which may lead to glioma cell survival, and it is regulated by its molecular marker protein—Beclin-1. However, glioma cells develop resistance for programmed cell death induction which causes decrease in the efficacy ratio of drugs used in standard treatment (Roos et al. 2007; 2. Brentnall et al. 2013, Jakubowicz-Gil et al. 2014). Therefore, continuous investigation of the molecular biology of gliomas is needed to study cancer nature more accurately and to find new possible treatment (Omuro et al. 2007).

It is known that brain cancers have developed many molecular mechanisms leading to cell survival responsible for their chemotherapy resistance. An example of such mechanism is the amplification of PI3K-Akt/PKB-mTOR pathway in glioma cells. This signaling cascade controls metabolism, proliferation, and motility in normal conditions, and it could be used by cancer cells for survival and inappropriate differentiation (Omuro et al. 2007). Therefore, inhibition of this molecular pathway may be considered as a molecular target in new therapy development. A specific inhibitor of phosphatidylinositol 3-kinase (PI3K) is LY294002, the morpholine synthetic derivative of natural flavonoid quercetin. This is the first generation of pan-PI3K inhibitor with anticancer activity in vitro through decreasing of cancer cell proliferation and increasing the apoptosis rate (Wee and Wang 2017). The other survival mechanism engaged in promotion of gliomas proliferation is a mitogenic Ras-Raf-MEK-ERK pathway. Such signal transmission may be blocked by sorafenib, a targeted cancer drug used in kidney, hepatocellular, and thyroid cancers with special affinity to Raf kinase. The interest of this drug rose after information about possible antiglioma activity (Lange et al. 2016; Wang et al. 2016). It inhibited new blood vessel formation and cell proliferation, which led to autophagy or apoptosis initiation in consequence. As it has been shown in former research, simultaneous application of sorafenib with quercetin is more effective than single application (Li et al. 2016; Jakubowicz-Gil et al. 2017). Therefore, the aim of the present study was to investigate for the first time the effect of single-targeted flavonoid derivative LY294002 in combination with sorafenib in the elimination of human glioma cells by PCD induction. Special attention was paid to the involvement of the expression of PI3K and Raf kinases in this process.

## Materials and methods

### Cells and culture conditions

Human anaplastic astrocytoma cells (MOGGCCM, European Collection of Cell Cultures) and human glioblastoma multiforme cells (T98G, European Collection of Cell Cultures) were grown in a 3:1 mixture of Dulbecco’s modified Eagle medium (DMEM) and Ham’s nutrient mixture F-12 (Sigma) supplemented with 10% fetal bovine serum (Sigma), penicillin (100 units/ml) (Sigma), and streptomycin (100 µg/ml) (Sigma). The cultures were kept at 37 °C in humidified atmosphere of 95% air and 5% CO_2_.

### Drug treatment

**Fig. 1:**
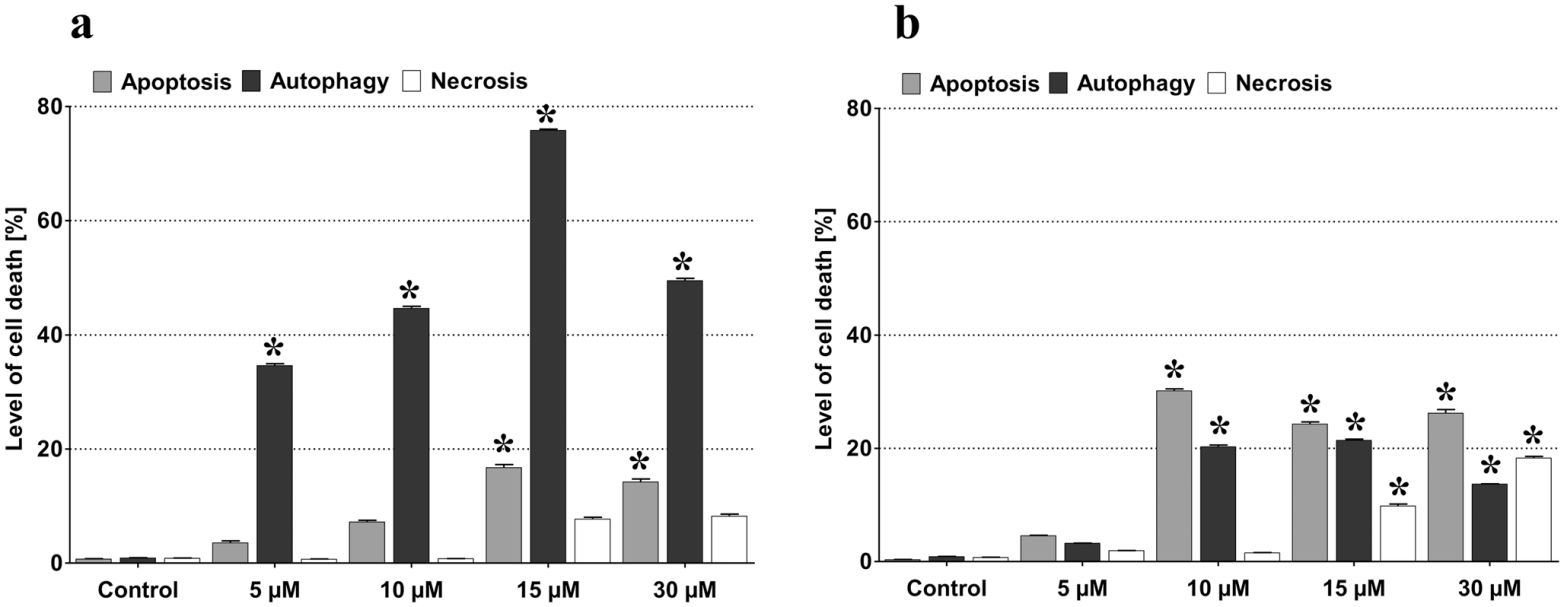
Effect of 24-h-long incubation of MOGGCCM (**a**) and T98G (**b**) cells with different (0–30 µm) concentration of LY294002. *P < 0.01

LY294002 (Sigma-Aldrich) and sorafenib (Nexavar, BAY 43–9006) were dissolved in DMSO. The drugs doses were chosen experimentally (in case of LY294002, 10 μM: Fig. 1) or based on previous experiments (sorafenib, 1 μM; Jakubowicz-Gil et al. 2017). Those concentrations of drugs were used in all experiments. MOGGCCM and T98G cells were treated with studied drugs separately or in combination (at the same time) for 24 h. As controls, cells were incubated with 0.01% of DMSO only. DMSO concentration was the same in all variants including controls and experiments.

### Microscopic detection of apoptosis, and necrosis with fluorochromes

Identification of apoptosis and necrosis was performed by a staining method with fluorescent dyes Hoechst 33342 (Sigma) and propidium iodide (Sigma). Control and treated cells cultured in chambered cell culture slide were stained with 1 µl of Hoechst 33342 and propidium iodide solution (in 2:1 proportion) (Sigma) by 5 min in 37 °C. After this time, the staining solution was replaced with PBS buffer with Ca^2+^ and Mg^2+^ ions. For morphological analysis of dead cells, a confocal microscope (Axiovert 200 M with scanning head LSM 5 PASCAL, Zeiss) was used. Typical morphological changes were observed. Cells exhibiting blue fluorescent nuclei (fragmented and/or with condensed chromatin) were interpreted as apoptotic, and cells with pink fluorescent nuclei were interpreted as necrotic. At least 1000 cells in randomly selected microscopic fields were counted under the microscope. Each experiment was performed in triplicate.

### Detection of acidic vesicular organelles with acridine orange

Autophagy level was evaluated by observing characteristic acidic vesicular organelles (AVOs) formed in the process. The organelles were staining with acridine orange (Jakubowicz-Gil et al. 2013; Jakubowicz-Gil et al. 2014). Control and treated cells cultured in chambered cell culture slide were stained with 1.25 µl of orange acridine (Sigma) by 15 min in 37 °C. After this time, the staining solution was replaced with PBS buffer with Ca^2+^ and Mg^2+^ ions. At least 1000 cells in randomly selected microscopic fields were counted under the microscope. Each experiment was performed in triplicate.

### Detection of mitochondrial membrane potential by flow cytometry

Mitochondrial membrane potential changes (Δψm) (MMP) in cells incubated with LY294002 and sorafenib were analyzed by staining with fluorochrome 3,3′-dihexyloxacarbocyanine iodide [DiOC6(3)] according to the method described previously (Bradford 1976). The stain accumulates in mitochondria at low concentration. The DiOC6(3) loss indicates disruption of mitochondrial inner transmembrane potential. Treated cells and control ones were incubated with fluorochrome for 20 min at 37 °C in the dark, washed three times with phosphate-buffered saline (PBS), and analyzed. Test was performed with the FacsCanto instrument (Becton Dickinson, San Jose, California, USA) in triplicate. The results were presented as the intensity of DiOC6(3) brightness.

### Caspase activity assay

Control and threated cells were analyzed for the activity of caspases 3, 8, and 9 with a SensoLyte®AMC Caspase Substrate Sampler Kit (AnaSpec) according to the manufacturer’s protocol. The fluorescence of 7-aminocoumarin (AMC) was inspected at *E*_*x*_*/E*_*m*_ = 354/422 nm in 96-well black microplates by 2030 Multilabel Reader Victor_TMx4_ (Perkin Elmer) microplate reader.

### Immunoblotting

Whole cell extracts were prepared by lysing cells in hot buffer containing 125 mM Tris–HCl pH 6.8, 4% SDS, 10% glycerol, and 100 mM dithiothreitol (DTT). The Bradford method was used for protein concentration measure (Bradford 1976). In total, 80 μg of proteins was separated by 10% SDS-PAGE (Laemmli 1970) and electroblotted onto Immobilon-P PVDF membrane (Sigma). Membranes were blocked with 1% low fat milk for 1 h and then incubated overnight with primary antibodies: mouse monoclonal antibody anti-PI3K, anti-Raf, anti-Beclin-1 and anti-caspase-3 (Santa Cruz Biotechnology, concentration 0.5 μg/ml). After three washes with PBS enriched with 0.05% Triton X-100 (Sigma), the membranes were incubated with secondary antibodies conjugated with alkaline phosphatase (AP) for 2 h. Proteins were detected with AP substrates: 5-bromo-4-chloro-3-indolylphosphate (BCIP) and nitro-blue tetrazolium (NBT) (Sigma) in *N*,*N*-dimethylformamide (DMF, Sigma). The results obtained were analyzed qualitatively on the basis of the band thickness, width, and color depth. The quantitative analysis of protein bands was performed using the ImageJ program. The data were normalized relative to β-actin (Sigma, working dilution 1:2000). Three independent experiments were performed.

### ELISA assay

The activity of Akt/PKB and ERK kinases was investigated with a AKT/ERK Activation InstantOne ELISA™ Kit (Invitrogen by Thermo Fisher Scientific) according to the manufacturer’s protocol. Control and treated cell lysates were incubated in 96-well plate with antibody cocktail, and after the washing steps, treated with 3,3′,5,5′-tetramethylbenzidine (TMB) colorimetric substrate and analyzed by 2030 Multilabel Reader Victor_TMx4_ (Perkin Elmer) microplate reader.

### Transfection with siRNA

The cells at a density of 2 × 10^5^ were incubated for 24 h at 37 °C in a CO_2_ incubator to reach 60–80% of confluence. After washing with a 3:1 DMEM/Ham’s F-12 mixture without serum and antibiotics, the medium was aspirated. The cells were overlaid with transfection probes containing 2 μl of specific anti-PI3K or anti-Raf small interfering RNA (siRNA) (Santa Cruz Biotech) and 2 μl of transfection reagent (Santa Cruz Biotech). After 5 h of incubation at 37 °C in a CO_2_ incubator, the medium was supplemented with medium containing 20% of fetal bovine serum and 200 μg/ml of antibiotics. Incubation for additional 18 h was performed. After changing the medium to the fresh normal growth one, such transfected cells were taken for further experiments.

### Statistical analysis

One-way ANOVA test followed by Dunnett’s multiple comparison analysis was used for statistical evaluation. P < 0.05 of data presented as mean ± standard deviation (SD) was taken as the criterion of significance. Validation of optimal dose selection of studied compounds was performed with Chou-Talalay’s test (Supplementary Files).

## Results

### The effect of LY294002 on apoptosis, autophagy, and necrosis induction in glioma cells

Cell death in the MOGGCCM and T98G cells after LY294002 treatment (5, 10, 15, and 30 µM) was estimated by staining methods using fluorochromes: propidium iodide, Hoechst33342, and orange acridine as described in “Material and methods.” As it is presented in Fig. 1a, 15 µM concentration of LY294002 seemed to be the most effective in programmed cell death induction in MOGGCCM cells, and autophagy was the dominant type of death, but unfortunately, it was accompanied by significant necrotic effect. In the case of 10 µM, autophagy reached 44.61% with no necrotic effect (Fig. 1a). In T98G cells, in turn, the most effective was 10 µM of LY294002, but in contrast to MOGGCCM, the apoptosis (Fig. 1b) was dominative (36%), accompanied by 20.26% of autophagy and no necrotic effect. Therefore, 10 µM concentration was chosen for further experiments.

### Cell death in MOGGCCM and T98G cells after simultaneous application of LY294002 and sorafenib

**Fig. 2:**
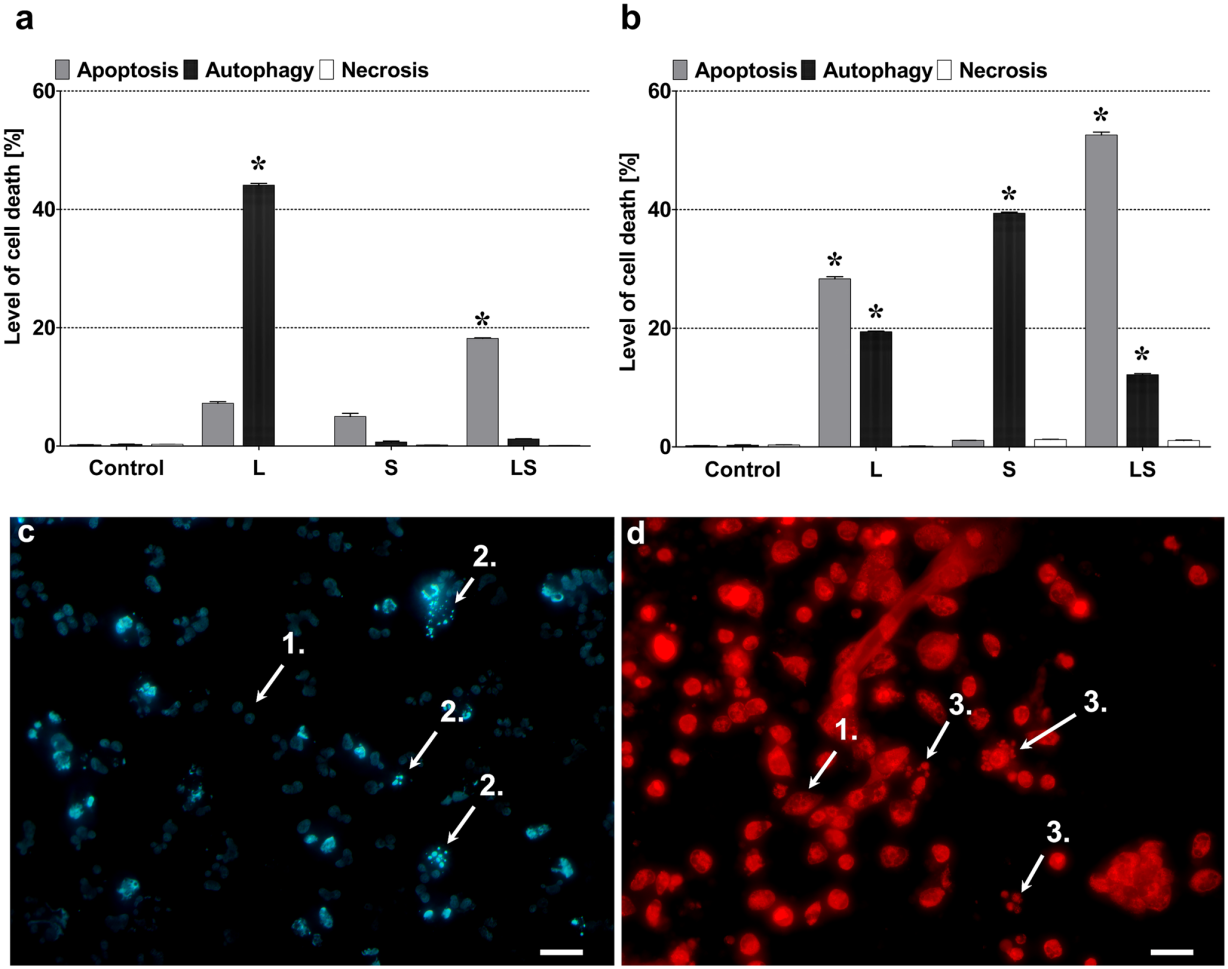
Evaluation of the level of apoptosis, necrosis and autophagy in MOGGCCM (**a**) and T98G (**b**, **c**, **d**) cells (scale bar—white line—50 µm) after 24 h sorafenib (1 µl) and LY294002 (10 µl) treatment. L LY294002, S sorafenib, LS LY294002 + sorafenib, *P < 0.01

On the basis of observation that combination therapy may have stronger anticancer potential than single drug application, AA and GBM cells were incubated with LY294002 and sorafenib at the same time (Fig. 2). Our results after simultaneous MOGGCCM cells treatment showed that apoptosis was the dominant type of death (about 20%), and the effect was more than doubled in comparison to the single drug application (Fig. 2a). It was correlated with low autophagic effect (about 3%) in contrast to single treatment. Similar observations were noticed in T98G cells after simultaneous treatment. The apoptosis was dominant and reach over 50% with significant decrease in the number of autophagic cells (about 10%) compared to single drug treatment (Fig. 2b).

### Effect of LY294002 and sorafenib on MMP

**Fig. 3:**
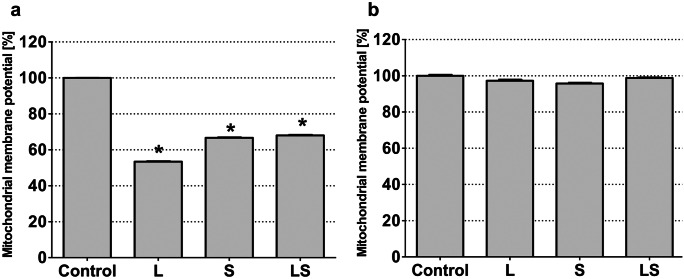
Analysis of mitochondrial membrane potential after LY294002 and sorafenib incubated MOGGCCM (**a**) and T98G (**b**) cells. L LY294002, S sorafenib, LS LY294002 + sorafenib, *P < 0.01

Anaplastic astrocytoma (Fig. 3a) and glioblastoma multiforme cells (Fig. 3b) were incubated with sorafenib and LY294002 separately and simultaneously and analyzed for the mitochondrial membrane potential to confirm the apoptotic type of death observed under the microscope. In MOGGCCM cells, both sorafenib and LY294002 applied alone or in combination scientifically decreased the MMP, but LY294002 was the most effective. In T98G line, none of inhibitors, applied alone or in combination, had any effect on the mitochondrial membrane potential, and its value was comparable to the control one.

### Evaluation of marker proteins upon LY294002 and sorafenib treatment

**Fig. 4:**
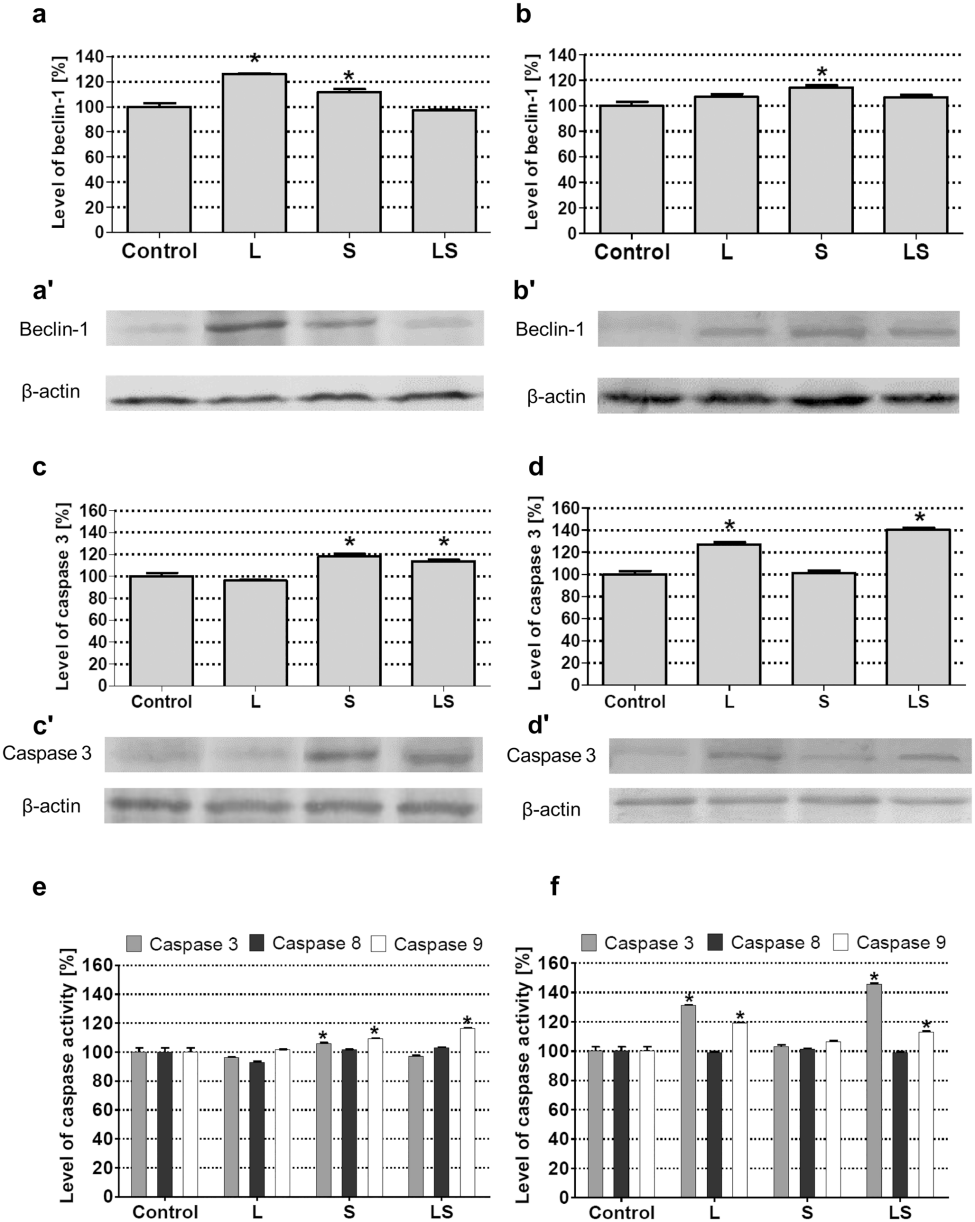
Evaluation of programmed cell death marker proteins expression: densitograms (**a**, **b**, **c**, **d**) and immunoblotting membranes (**a′**, **b′**, **c′**, **d′**) of beclin-1 (**a**, **b**) and caspase 3 (**c**, **d**) and activity: caspases 3, 8, and 9 (**e**, **f**) in MOGGCCM (**a**, **c**, **e**) and T98G (**b**, **d**, **f**) cells. L LY294002, S sorafenib, LS LY294002 + sorafenib, *P < 0.01

Apoptosis and autophagy at the molecular level are characterized by changes in the expression of marker proteins. In our experiments, we studied the level of pro-autophagic Beclin-1 and pro-apoptotic caspase 3 expression as well as the activity of caspases 3, 8, and 9. In the case of Beclin-1 in MOGGCCM cells (Fig. 4a), significant increase in the protein expression was observed after separate LY294002 and sorafenib incubation. In T98G, overexpression of autophagy marker was observed in all experimental variants, but only sorafenib was significant (Fig. 4b). According to apoptotic marker, the level of caspase 3 expression in MOGGCCM cells was increased after sorafenib treatment only (Fig. 4c). The LY294002 alone and/or in combination with sorafenib had no significant influence on the protein expression. In T98G cells, single and simultaneous application of LY294002 and sorafenib increased the level of caspase 3 expression, but in the case of sorafenib alone, the effect was not significant (Fig. 4d).

It is well known that not only the level of expression, but also the activity of caspases has important impact on apoptosis initiation. In our experiment conducted on MOGGCCM cells, sorafenib caused the significant increase of caspase 3 and 9 activity while LY294002 caspase 9 (Fig. 4e). In glioblastoma multiforme cells, only LY294002 alone or in combination with sorafenib stimulated the activity of caspases 3 and 9.

**Fig. 5:**
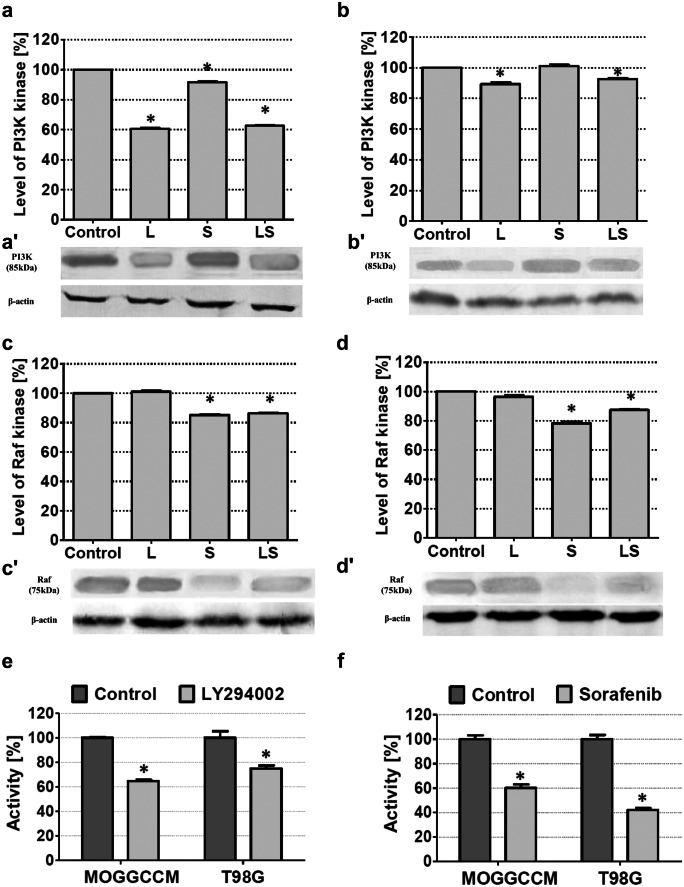
Estimation of PI3K (**a**, **b**) and Raf (**c**, **d**) kinases expression [densitograms (**a**, **b**, **c**, **d**) and immunoblotting membranes (**a′**, **b′**, **c′**, **d′**)] and AKT (**e**) and ERK (**f**) activity in MOGGCCM (**a**, **c**) and T98G (**b**, **d**) cells after studied drugs application. L LY294002, S sorafenib, LS LY294002 + sorafenib, *P < 0.01

Quantitative and qualitative analyses of the immunoblots revealed that in MOGGCCM and T98G cells, LY294002 and sorafenib successfully diminished the level of PI3K and Raf expression respectively (Fig. 5). The inhibitory effect was also observed after simultaneous drugs application (Fig. 5). The inhibitory effect of LY294002 and/or sorafenib also diminished the activity of downstream enzymes of PI3K-Akt/PKB-mTOR and Ras-Raf-MEK-ERK pathways like Akt/PKB (in case of LY294002 action exclusively) and ERK (by sorafenib activity only) (Fig. 5e, f).

### Blocking of PI3K and Raf expression in MOGGCCM and T98G cells and its role in cell death induction

**Fig. 6:**
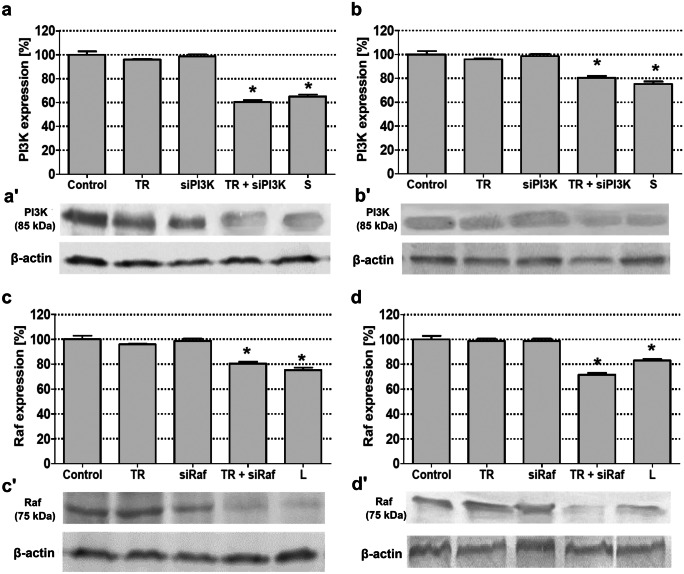
The effect of blocking PI3K (**a**, **b**) and Raf (**c**, **d**) expression [densitograms (**a**, **b**, **c**, **d**) and immunoblotting membranes (**a′**, **b′**, **c′**, **d′**)] with specific siRNA in MOGGCCM (**a**, **c**) and T98G (**b**, **d**) cells. TR transfection reagent, L LY294002, S sorafenib, *P < 0.01

**Fig. 7:**
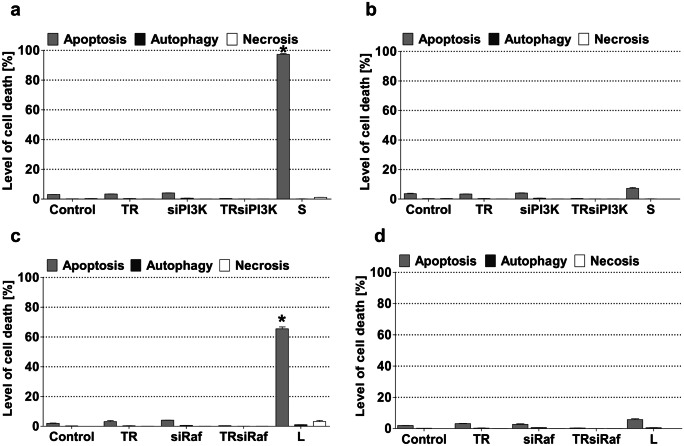
Examination of programmed cell death level in MOGGCCM (**a**, **c**) and T98G (**b**, **d**) after blocking expression with specific siRNAs, TR transfection reagent, L LY294002, S sorafenib, *P < 0.01

To receive a direct proof of the involvement of PI3K and Raf expression in the resistance of gliomas to programmed death and to confirm inhibitory potential of LY294002 and sorafenib, the expression of the studied kinases in the MOGGCCM and T98G cells was blocked using the transfection assay with specific small interfering RNA (siRNA) (Fig. 6). Immunoblots of transfected cells revealed that siRNA significantly limited the expression of PI3K and Raf in both cell lines. Such effect was sustained after the incubation with LY294002 and sorafenib. Microscopic analysis of the morphology of transfected MOGGCCM and T98G cells showed that incubation with LY294002 and sorafenib was effective in cell death initiation. Anaplastic astrocytoma cells turned out to be more sensitive to elimination by programmed cell death than glioblastoma multiforme one. The incubation of siPI3K transfected MOGGCCM line with sorafenib resulted in the initiation of apoptosis in over 90% of the cells. In the same cell line transfected with siRaf, LY294002 application induced this type of death in over 60% of AA population. Surprisingly, transfected T98G cells turned out to be very resistant for programmed cell death initiator after the LY294002 and sorafenib application, and the number of dead cells did not exceed 10%. The inhibition of PI3K or Raf expression by specific siRNA in the MOGGCCM and T98G cells did not increase the level of autophagy or necrosis, and apoptosis was dominative (Fig. 7).

## Discussion

Malignant gliomas such as anaplastic astrocytoma or glioblastoma multiforme are considered as one of the most malignant cancers, incurable so far. Even standard treatment involving surgical resection followed by chemo- or radiotherapy only prolongs patient life and slightly improves its comfort (Johnson and Galanis 2014, Tran and Rosenthal 2010). The currently used cytostatics are not effective, that is why the alternative treatment is needed (Wu et al. 2016). The aggressive character of gliomas and their resistance for currently available treatment resulted from overexpression of the intracellular survival pathways: PI3K-Akt/PKB-mTOR and Ras-Raf-MEK-ERK. This upregulation of signaling cascades has been proven to take part in amplification of glioma proliferation rate and avoiding the programmed cell death induction. That is why the disruption in these signaling transductions seems to be key molecular target of modern therapies. It is known that combination therapy is more effective in cancer cell elimination (Xu et al. 2015). It is based on simultaneous application of two or more different drugs (Wang et al. 2015; Lien et al. 2016, Majewska and Szeliga 2017). The best strategy is to combine two drugs with different mechanisms of action that would lead to the elimination of cancer cells by programmed cell death initiation. Our earlier experiments conducted on MOGGCCM and T98G lines revealed that sorafenib and natural flavonoid—quercetin—were very effective in programmed cell death induction. However, quercetin is a non-specific protein kinase enzyme inhibitor (Carvalho et al. 2017). LY294002 seems to be better element of combination therapy, because it was designed as a morpholine synthetic derivative of quercetin with precise mechanism of action directed toward abolishing PI3-kinase enzymatic activity. It affects cell proliferation, through the PI3K-Akt/PKB-mTOR pathway, and may lead to PCD induction in cancer cells (Janku 2017, Jinkan et al. 2018). Sorafenib, in turn, used as standard treatment against liver or kidney cancer, diminishes the activity of serine/threonine kinases including Raf kinase (Cervello et al. 2012, Zhao and Adjei 2015). Therefore, the aim of the present study was to evaluate the combined effect of sorafenib and LY294002 toward programmed cell death induction in human glioma cells. The inhibitory activity of LY294002 reduced LN229 (glioblastoma) and U251 (astrocytoma) cells proliferation and invasiveness in vitro and impacted the expression of multiple survival pathways like PI3K-Akt/PKB-mTOR (Jane et al. 2006). Research conducted on U87 cells (GBM) has shown that the application of LY294002 decreased the invasive capability of glioma cells which was correlated with downregulation of PI3K expression and Akt activity, which led to apoptosis induction in consequence. Similar results were observed in LY294002-treated nasopharyngeal carcinoma cells (Hunguo et al. 2010, Chen et al. 2012). In osteosarcoma’s cancer stem-like cells, LY294002 treatment corresponded with G0/G1 cell cycle arrest (Gong et al. 2012). Moreover, it was discovered that LY294002 initiated apoptosis through intrinsic and extrinsic pathways, which was associated with significant increase of caspases 9 or 8 activity respectively (Pathania et al. 2013). Our experiments showed that almost 45% of anaplastic astrocytoma cells after LY294002 incubation were eliminated on the way of autophagy. On the other hand, in LY294002-tretaed glioblastoma multiforme cells, the apoptosis was the dominant type of death. The autophagy induction in AA cells was correlated with the increase of its cellular marker expression—Beclin-1. In the case of apoptosis induction in GBM cells, the decrease of mitochondrial membrane potential was observed with the significant increase of caspases 3 and 9 activity, which may suggest that this type of programmed death was conducted by intrinsic pathway. In AA cells where autophagy was dominant, the involvement of caspases was not significant. LY294002 also appeared as good inhibitor of PI3K expression in both cell lines which was correlated with reduced Akt activity at the same time, which confirmed that LY294002 is a potent PI3K-Akt/PKB-mTOR pathway inhibitor.

Cytotoxic potential of sorafenib against cancer cells by inhibitory effect on Raf kinase has been widely described in the literature (Huang et al. 2010; Brenthall et al. 2013). It was observed that sorafenib-induced apoptosis was based on mitochondrial membrane disruption, cytochrome c release to the cytoplasm, caspases 9 and 3 activation, and cell death through intrinsic pathway (Huang et al. 2010). On the other hand, in different studies, scientists observed that sorafenib-induced apoptosis proceeded with toll-like receptor involvement and caspase 8 activation (Brenthall et al. 2013). There are also reports that suggested that the dominant type of death in cancer cells after sorafenib incubation is autophagy. It is with agreement with our previous studies where GBM cells were mainly eliminated by autophagy induction (Jakubowicz-Gil et al. 2014; Carvalho et al. 2017; Jakubowicz-Gil et al. 2017). Our present experiments confirmed these correlations. In T98G cells, sorafenib induced mainly autophagy, which was correlated with increase of Beclin-1 expression. At the moment, the autophagy is a controversial process in context of elimination of cancer cells. It is believed that it may represent the survival mechanism and determines cancer resistance for treatment. That is why the apoptosis is a more desirable mechanism of cancer cell elimination (Jakubowicz-Gil et al. 2014; Jakubowicz-Gil et al. 2017). On the other hand, our studies showed that in MOGGCCM cell incubation with sorafenib induced mainly apoptosis, and it was accompanied by significant increasing in caspases 3 and 9 activity and decreasing in mitochondrial membrane potential. Those results suggest that sorafenib induced intrinsic apoptotic pathway in astrocytoma cells. The inhibitory effect of sorafenib against Raf kinase expression was proved by immunoblotting and was significantly decreased in both cell lines. Moreover, in AA and GBM sorafenib-treated cells, the significant decrease in ERK activity was observed. This gives a reason to believe that sorafenib is a good Ras-Raf-MEK-ERK pathway inhibitor.

In our studies estimated for the first time, the combined effect of LY294002 and sorafenib on programmed cell death induction on glioma cells was evaluated. It is known that single drug application is less effective than combination therapy. The studies with sorafenib combined with other agents such as rottlerin, tipifarnib, quercetin or temozolomide, and their effectiveness in elimination of glioma cells proved such observations (Den et al. 2012; Gong et al. 2012; Jakubowicz-Gil et al. 2014; Jane et al. 2006; Jakubowicz-Gil et al. 2017; Nan et al. 2017; Ngihiemphu et al. 2017). In our study, we have observed that LY294002 and sorafenib (LS) appeared to be an effective drug couple that eliminated T98G and MOGGCCM glioma cells upon programmed cell death, and the apoptosis was the dominant type of death. What was more interesting, in both cell lines after LS treatment is that the level of autophagy was significantly reduced in comparison to single drug application. It was correlated with the increase of caspase 3 expression and activity in T98G cells. Interestingly, the mitochondrial membrane potential was not significantly involved in this process. Surprisingly, in apoptotic MOGGCCM cells, increase in caspase 3 expression as well as caspases 3 and 9 activity was not significant. It may be explained by other observations, suggesting that apoptotic intrinsic pathway proceeded without activation of caspase 3, which may be induced by caspase 7 activation (Brenthall et al. 2013). Moreover, in anaplastic astrocytoma as well as glioblastoma multiforme, combination of LS significantly decreased the level of PI3k and Raf expression.

To receive a direct proof of the involvement of PI3K and Raf in the resistance of glioma cells to cell death initiation, we decided to block these kinases with specific siRNA. In both transfected MOGGCCM and T98G, cells apoptosis was the most dominant type of death, but in GBM cells, it was not on significant level. It may suggest the glioblastoma multiforme is more resistant to siRNA treatment. That proved that the direct inhibition of PI3K-Akt/PKB-mTOR and Ras-Raf-MEK-ERK pathway is not sufficient to eliminate GBM cells on the way of programmed death. It seems that the studied drugs pose wider spectrum of action, which allowed more efficient apoptosis induction.

## Conclusion

Taken together, our studies showed the PI3K-Akt/PKB-mTOR and Ras-Raf-MEK-ERK pathways are mainly responsible for chemoresistance of glioma cells. Blocking the signal transmission through these enzymes by new drug combination, LY294002 and sorafenib led to the effective elimination of cancer cells by apoptosis without significant autophagic or necrotic effect.

Our data may form the background for modern molecular targeting therapy which may form a base for further studies on new treatment strategy against human glioma cells.

## Supplementary Information

Below is the link to the electronic supplementary material.

Supplementary file1 (DOCX 76 KB)

Supplementary file2 (DOCX 75 KB)

## Abbreviations

AA: Anaplastic astrocytoma
AKT: Protein kinase B (PKB)
GBM: Glioblastoma multiforme
ERK: Extracellular signal-regulated kinase
MEK: Mitogen-activated protein kinase kinase (MAPKK)
mTOR: Mammalian target of rapamycin
PCD: Programmed cell death
PI3K: Phosphatidylinositide 3-kinase
Raf: Raf kinase
RTK: Receptor tyrosine kinase
TMZ: Temozolomide
