# Supplementary material for: LY294002 and sorafenib as inhibitors of intracellular survival pathways in the elimination of human glioma cells by programmed cell death
Source: Cell Tissue Res. 2021 Jul 8;386(1):17–28. doi: 10.1007/s00441-021-03481-0 (PMC8526469; doi:10.1007/s00441-021-03481-0)
Supplement: Supplementary file 2 — Supplementary file2 (DOCX 75 KB) [file 441_2021_3481_MOESM2_ESM.docx]

**
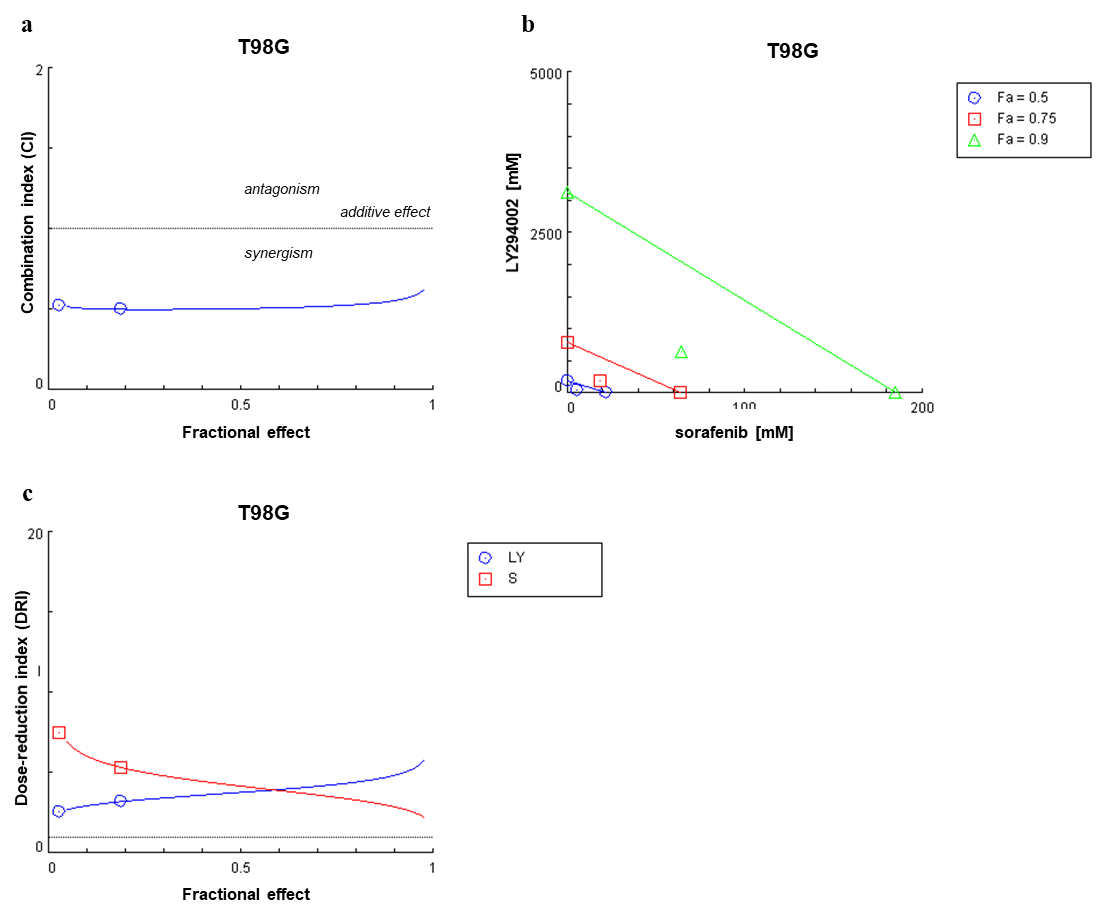
Fig.2**

**Fig. 2** LY294002 and sorafenib combination treatment. (a) Combination index (CI) plot: The combination index is plotted as a function of Fa (fractional effect). (b) Isobologram for combination: Classic isobologram at IC50, IC75, and IC90. (c) the Fa-DRI (dose reduction index) plot (Chou-Martin plot). Combination index (CI) and dose reduction index (DRI) were calculated according to the method of Chou and Talalay using the Compusyn software. CI<1, CI=1 and CI>1 indicate synergistic effect, additive effect, and antagonistic effect, respectively. The DRI represents the fold reduction of TMZ as a result of synergistic combination compared to the concentration of drug treatment alone needed to reach the same effect.

These results showed the efficacy of LY294002 to improve sorafenib-mediated cytotoxicity by reducing its concentration. Therefore, LY294002 in combination with sorafenib have
a synergistic effect on the apoptosis induction of glioma cells.
